# Know Your Enemy: *Piscirickettsia salmonis* and Phage Interactions Using an In Silico Perspective

**DOI:** 10.3390/antibiotics14060558

**Published:** 2025-05-30

**Authors:** Carolina Ramírez, Jaime Romero

**Affiliations:** Laboratorio de Biotecnología de Alimentos, Instituto de Nutrición y Tecnología de los Alimentos (INTA), Universidad de Chile, El Líbano 5524, Santiago 7830489, Chile; carolina.ramirez.saavedra@gmail.com

**Keywords:** *Piscirickettsia salmonis*, salmonid rickettsial septicemia (SRS), phage, prophage, aquaculture

## Abstract

**Background:** Aquaculture faces significant challenges due to bacterial infections, particularly *Piscirickettsia salmonis*, leading to extensive antibiotic use and raising concerns about antimicrobial resistance. In this context, bacteriophages and bacterial defense systems play a critical role in the evolutionary dynamics of *P. salmonis*. **Objective.** This study aimed to investigate the genomic landscape of prophage regions and antiphage defense systems in Piscirickettsia salmonis to better understand their co-evolutionary dynamics and explore their potential role in alternative disease control strategies for aquaculture. **Methods:** We analyzed 79 genomes of *Piscirickettsia salmonis* using bioinformatic tools to identify and characterize prophage regions and antiphage defense systems. **Results:** At the chromosomal level, 70% of the strains contained prophage regions, with a total of 92 identified regions, most of which were classified as intact. At the plasmid level, 75% of plasmids carried prophage regions, with a total of 426 identified regions, predominantly associated with *Escherichia phage RCS47*, *Burkholderia phage Bcep176*, and *Enterobacteria phage mEp235*. Prophage regions were enriched in transposases, head proteins, tail proteins, and phage-like proteins. The analysis of antiphage defense systems revealed that *P. salmonis* predominantly harbors dGTPase, AbidD, and SoFIC at the chromosomal level, whereas MazEF was the most frequent system in plasmids. A strong positive correlation was found between the number of prophage regions and defense systems in chromosomes (ρ = 0.72, *p* = 6.3 × 10^−14^), while a weaker correlation was observed in plasmids. These findings highlight the complex interplay between *P. salmonis* and its bacteriophages, with implications for disease control in aquaculture. **Conclusions:** Overall, these insights into the prophage and defense system dynamics provide potential avenues for developing alternative strategies to combat *P. salmonis* infections and reduce reliance on antibiotics in aquaculture systems.

## 1. Introduction

*Piscirickettsia salmonis* is a Gram-negative, facultative intracellular bacterium and the etiological agent of Piscirickettsiosis, also called Salmonid Rickettsial Septicemia or SRS, previously Salmonid Rickettsial Syndrome [1]. It has been stated that SRS remains the main systemic bacterial disease affecting Chilean salmon farming, causing high mortality and resulting in estimated annual losses of up to USD 700 million [2,3]. *P. salmonis* causes systemic infection in Atlantic salmon and leads to septicemia, which affects the brain, heart, liver, kidney, spleen, intestine, and ovaries [3]. Although it has been associated with high mortality rates in farmed salmon, recent findings challenge its classification as an obligate pathogen [3]. Genomic and ecological evidence indicates that *P. salmonis* can survive in seawater and cell-free media, is frequently detected in healthy fish, and harbors incomplete or disrupted virulence-related gene clusters, such as a degenerate Dot/Icm secretion system, suggesting that its pathogenicity may be conditional rather than intrinsic [3,4]. Its pathogenicity appears conditional, likely triggered by specific host or environmental stressors such as dysbiosis or immunosuppression [3].

Currently, there are more than 30 registered commercial vaccines in use to control this pathogen [5]. However, available vaccines against SRS in Chile have poor effectiveness. Differences may be due to different vaccination protocols used by the Chilean salmon industry, including adjuvanted vaccines for primary intraperitoneal immunization, which in some cases can be followed by an oral boost [6]. However, the efficacy of each of the vaccine formulations is not consistent, mainly due to the contradictory results obtained with protocols based on bacterins. Furthermore, it is not known whether vaccination will grant the desired long-term protection. In summary, available vaccines have not demonstrated the expected efficacy under field conditions [5,6,7].

The use of antibiotics in the Chilean aquaculture industry has steadily increased in correlation with intensified salmonid production. Data from the National Fisheries and Aquaculture Service of Chile, known as SERNAPESCA, confirm that Atlantic salmon cultures receive the largest amount of antibiotics with respect to the other species in cultivation [8]. In 2015 the usage of antibiotics reached its highest level, with 630 g per harvested ton. Since then, an annual trend to reduce the use of antimicrobials was observed; however, in 2023, the usage increased and reached 310 g per harvested ton [8]. Furthermore, more than 90% of antibiotics (oxytetracycline and florfenicol) administered in the control of SRS are used in seawater. Nevertheless, antibiotic consumption has been nearly halved compared to peak levels, demonstrating significant progress toward more sustainable disease management strategies in Chilean aquaculture [9,10].

Bacteriophages have been investigated as antimicrobial agents to control pathogenic bacteria in various fields, including aquaculture [11,12,13]. Research has reported multiple instances of phage applications targeting pathogenic vibrios, which represent a significant challenge in aquaculture [11,12,13]. Furthermore, the bacteriophage-based product Curtus has been specifically developed to combat *Yersinia* infections in aquaculture settings, offering a targeted and environmentally friendly approach to pathogen management and fish health improvement [14]. Transmission electron microscopy revealed the presence of phage-like particles associated with some *P. salmonis isolates*. This observation, made after purification through differential centrifugation and a 30% Percoll gradient, suggests the potential existence of bacteriophages interacting with *P. salmonis* [15]. However, despite these applied advances, the genomic basis of such interactions, particularly the prevalence of prophages and antiphage defense systems in key pathogens like *P. salmonis*, remains underexplored. Interactions between bacteriophages and their bacterial hosts are central to microbial ecology and evolution, shaping genome architecture and driving the emergence of complex defense and counter-defense systems [16,17]. These molecular arms races not only influence horizontal gene transfer and virulence but are also critical for the rational design of phage-based biocontrol strategies.

This study aims to enhance our understanding of the interactions between *P. salmonis*, a facultative intracellular pathogen, and its corresponding bacteriophages through genomic analysis, with a particular emphasis on bacterial defense mechanisms [16,17]. For this purpose, we used a publicly available *P. salmonis* genome sequence downloaded from the NCBI repository. Given the unique intracellular lifestyle of *P. salmonis*, elucidating these relationships could provide valuable insights into host–pathogen dynamics and contribute to the development of innovative strategies for controlling SRS while reducing reliance on antibiotics [18].

## 2. Results

### 2.1. Prophage Regions in P. salmonis Strains

**Figure 1:**
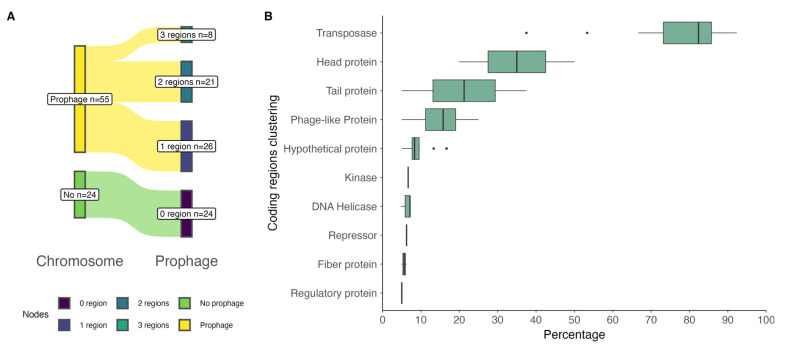
Prophage regions found at the chromosomal level in *P*. *salmonis* strains: (**A**) Number of prophage regions per chromosome (genome): green indicates no prophage detected in 24 strains; yellow indicates the presence of prophages in 55 strains; these were grouped based on the number of prophage regions detected. (**B**) Percentage clustering of coding regions (CDS), according to blastp results, contained in the prophage regions.

We analyzed the presence of prophage regions in 79 genomes of *P*. *salmonis* strains available in the NCBI repository. PHASTEST results revealed that, at the chromosomal level, approximately 70% of the *P*. *salmonis* strains (55 out of 79) had prophage regions. Of these strains, the majority had one or two prophage regions, and a smaller number had three regions (Figure 1A). A total of 92 prophage regions were found, 91 of which correspond to intact regions according to the classification assigned by PHASTEST, ranging in length from 13.8 Kb to 48 Kb, and one questionable region with a length of 7.2 Kb (Supplementary Material Table S1). In these regions, the most common phage found was *Escherichia* phage RCS47 (NC_042128).

A total of 1467 coding sequences (CDSs) were identified in the prophage regions. Per region, these CDSs corresponded mostly to transposases, with a median of 82% (IQR = 12.5%), followed by head protein, tail protein, and phage-like protein (Figure 1B). Details of the predicted CDSs, top blastp hits, and significant E-values are given in Table S2. See also Figure S1.

**Figure 2:**
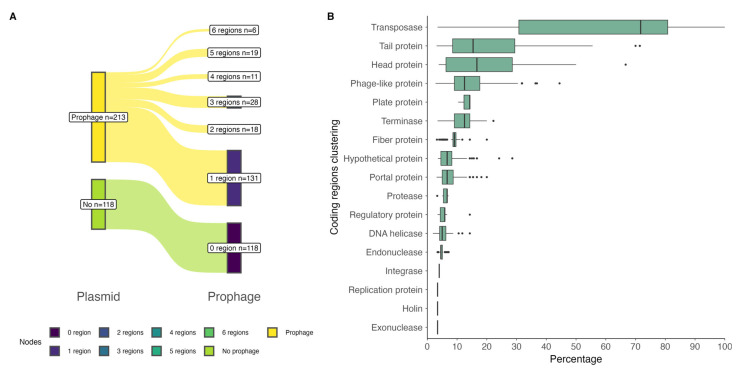
Prophage regions found at the plasmidial level in *P*. *salmonis* strains: (**A**) Number of prophage regions per plasmid: green indicates no prophage detected in 118 plasmids; yellow indicates the presence of prophages in 213 plasmids; these were grouped based on the number of prophage regions detected. (**B**) Percentage clustering of coding regions, according to blastp results, contained in the prophage regions.

At the plasmidial level, of the total 79 strains analyzed, 78 carried 331 plasmids, bringing a median of 4 plasmids per strain (Supplementary Material Table S3). The PHASTEST results showed that 213 plasmids, out of a total of 331, had prophage regions; these regions were present in 75% (median, IQR = 25%) of plasmids per strain (Supplementary Material Table S4). A total of 426 prophage regions were found in the 213 plasmids, of which 92.5% (394 regions) corresponded to regions classified as intact, 6.6% as questionable, and 0.94% as incomplete. Most of the plasmids contained one prophage region, followed by three and five regions (Figure 2A). The most common phages found in these regions corresponded to phages described against *Escherichia* (phage RCS47, NC_042128), *Burkholderia* (phage Bcep176, NC_007497), and *Enterobacteria* (phage mEp235, NC_019708).

A total of 7601 coding sequences (CDSs) were identified in the prophage regions. Per region, these CDSs were grouped into 17 protein types, of which transposases were the majority, with a median of 71% (IQR = 50%), followed by tail protein, head protein, and phage-like protein (Figure 2B). Details of the predicted CDSs, top blastp hits, and significant E-values are given in Table S5. See also Figure S1.

### 2.2. Defense System Diversities Across P. salmonis Strains

**Figure 3:**
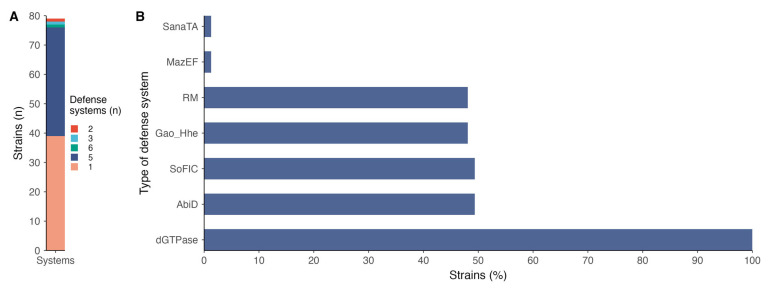
Defense systems in chromosomes of *P*. *salmonis* strains: (**A**) number of defense systems found at the chromosome level; (**B**) types of defense systems found in chromosomes.

Analysis of antiphage defense systems in *P*. *salmonis* revealed that mainly one or five defense systems are harbored at the chromosomal level, representing about 49% and 47% of strains, respectively (Figure 3A). The types of systems encountered were mainly represented by dGTPase, present in 100% of the strains, followed by AbidD (49.37%), SoFIC (49.37%), Gao-Hhe (48.10%), RM (48.10%), and underrepresented MazEF and SanaTA (Figure 3B; Supplementary Material Table S6).

**Figure 4:**
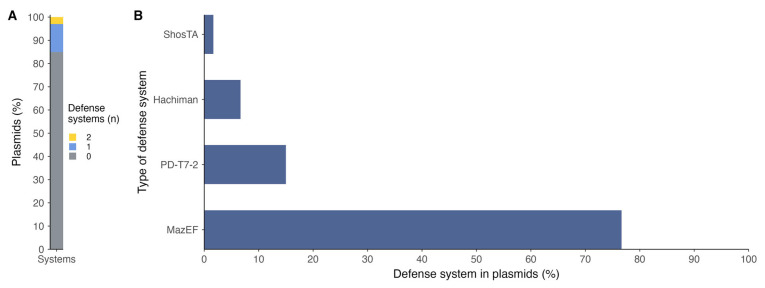
Defense systems in plasmids of *P*. *salmonis* strains: (**A**) number of defense systems in plasmids; (**B**) types of defense systems found in plasmids.

Of the total genomes analyzed, 78 strains contained plasmid genomic information, of which 37 comprised plasmidial defense systems. Of the total number of plasmids, only 15% contained antiphage defense systems, of which the majority presented only one system per plasmid (Figure 4A). The types of systems found in the plasmids were differently conformed in relation to those found in the chromosomes; in the case of plasmids, the majority type was MazEF (Figure 4B; Supplementary Material Table S7), which was present in a small proportion at the chromosome level.

**Figure 5:**
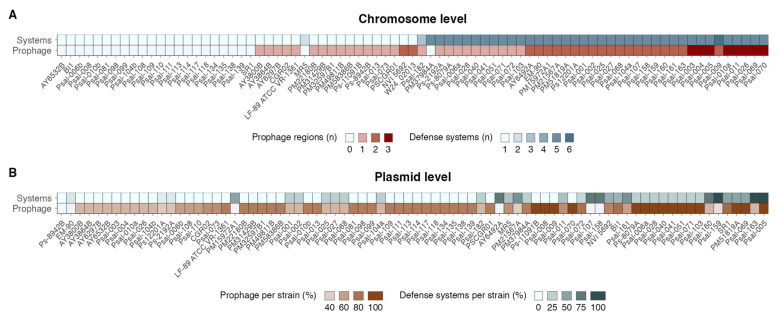
Summary of prophage regions and defense systems contained in chromosomes (**A**) and plasmids (**B**) per *P*. *salmonis* strain.

The number of prophage regions and the number of defense systems in the chromosomes of *P*. *salmonis* strains presented a high positive correlation (Spearman’s rho = 0.72, *p* = 6.3 × 10^−14^), indicating that the higher the number of prophage regions, the higher the number of antiphage systems present in the strains (Figure 5A). At the plasmid level, there is also a significant positive correlation between the number of prophage regions and defense systems; however, in this case, this correlation is low (Spearman’s rho = 0.22, *p* = 6.5 × 10^−5^). This is clear when visualizing the proportion of plasmids with prophage regions and defense systems per strain, where no trend is observed between these two variables (Figure 5B), as observed at the chromosomal level.

## 3. Discussion

Intracellular bacteria, which can survive within eukaryotic cells, are major causes of severe infections and contribute to high global mortality rates [18]. The intracellular environment provides protection against external threats, but bacteria must overcome harsh conditions such as acidic pH, lytic enzymes, and limited nutrients. While antibiotics remain the primary treatment, their effectiveness is limited by poor intracellular penetration and the increasing threat of antimicrobial resistance [18,19]. Bacteriophages (phages) offer a promising strategy. Evidence suggests that phages can enter both phagocytic and non-phagocytic eukaryotic cells while retaining viability to eliminate intracellular bacteria [19,20].

Prophages play a fundamental role in lysogenic conversion, a process where a temperate bacteriophage integrates into the bacterial genome and forms a prophage. This integration is a key mechanism for bacteria to acquire accessory traits [20,21]. Prophage sequences can contribute up to 20% of a bacterial genome, with variations between species and strains, and it is estimated that approximately 25% of all bacteriophages on Earth exist as prophages [20]. Prophages can be vertically transmitted and excised as plasmids, facilitating horizontal gene transfer and bacterial adaptation.

Prophage regions were commonly detected across the plasmids, with most classified as intact and varying in number per plasmid (Supplementary Material Table S4, Figure 2A). These findings suggest that the presence of prophage regions within plasmids may contribute to the horizontal transfer of genetic material, enhancing bacterial adaptability [21,22]. The enrichment of phage-related genes, including transposases and structural components, indicates potential roles in genome plasticity and bacterial evolution [23,24]. Additionally, the widespread occurrence of prophage elements across diverse plasmid backgrounds highlights their influence on shaping bacterial genomic content and promoting the dissemination of advantageous traits [25].

MazEF was the most common antiphage defense mechanism found in plasmids harbored by *P. salmonis*. MazEF is a toxin–antitoxin (TA) system, which serves as a bacterial defense mechanism against bacteriophage infection [26]. It consists of two components: MazE, a labile antitoxin, and MazF, a stable toxin [27]. Under normal conditions, MazE neutralizes the toxic effects of MazF. However, during phage infection or cellular stress, the antitoxin is degraded, allowing MazF to inhibit cell growth by cleaving RNA and shutting down essential cellular processes. This prevents the phage from replicating and spreading. The frequent presence of MazEF systems in *P. salmonis* plasmids may be attributed to their role in enhancing bacterial survival under phage attack. *P. salmonis* also activates the expression of the MazEF toxin–antitoxin operon during the initial stages of biofilm formation, likely as a mechanism to enhance survival under stressful conditions in marine environments [28]. Plasmids often carry beneficial traits that increase host adaptability, including defense mechanisms like MazEF [29]. Additionally, plasmids are highly mobile genetic elements, making them effective vectors for spreading TA systems across bacterial populations. This widespread distribution provides a selective advantage by reducing phage propagation, especially in environments where bacteriophages are prevalent.

The widespread presence of prophage regions in approximately 70% of *P. salmonis* strains highlights their potential role in the species’ genomic architecture and adaptation [30]. The predominance of 1–2 prophage regions per genome, with a few strains harboring three, indicates a relatively conserved prophage load across the population, possibly reflecting selective pressure to maintain beneficial elements. A striking feature of the prophage regions was the high abundance of transposases, with a median representation of 82% per region. This strong enrichment implies a significant contribution to genomic mobility and plasticity, which may facilitate rapid adaptation in dynamic environments, such as host-associated or marine settings [30,31]. The mobilization potential also raises the possibility of reshuffling genetic elements that could alter virulence or resistance traits [32].

The frequent detection of *Escherichia* phage RCS47 across diverse strains points toward a conserved acquisition event or selective maintenance of this prophage, potentially due to advantageous cargo genes [21]. This pattern suggests that certain prophages may offer evolutionary benefits to *P. salmonis*, promoting their retention over time [33]. Furthermore, the diversity of coding sequences within the prophage regions, including head and tail proteins and phage-like genes, supports the idea that these elements are more than genomic remnants [34]. Their presence may influence host physiology, modulate phage–bacteria interactions, or play a role in inter-microbial competition. Collectively, these findings underscore the importance of prophages as dynamic contributors to *P. salmonis* genome evolution and ecological fitness [35].

In *P. salmonis*, a variety of antiphage defense systems were identified, reflecting a complex and layered strategy to counter viral infections. The dGTPase system, present in 100% of the strains analyzed, plays a fundamental role by hydrolyzing intracellular deoxyguanosine triphosphate (dGTP), thereby disrupting DNA replication processes [36]. This depletion of nucleotides impairs both host and phage replication but serves as an effective means to block phage proliferation at an early stage, highlighting its role as a core, conserved line of defense [37,38]. The AbiD system, found in nearly half of the strains, belongs to the broader class of abortive infection (Abi) mechanisms. It operates by triggering programmed cell death or growth arrest upon detection of phage invasion, effectively sacrificing the infected cell to protect the bacterial community and prevent phage amplification, a form of altruistic defense common in high-density or biofilm-associated populations [39,40].

SoFIC (Suppressor of Filamentation Induced by cAMP), also present in ~49% of strains, is a more recently described system that appears to inhibit phage replication by interfering with vital cellular functions such as transcription or translation, although its precise mode of action remains under investigation [41]. DNA viruses consume large quantities of deoxynucleotides (dNTPs) when replicating, and Tal et al. [41] have identified a family of defensive bacterial deoxycytidine triphosphate (dCTP) deaminase proteins that convert dCTP into deoxyuracil nucleotides in response to phage infection. Its moderate prevalence in *P. salmonis* suggests it may serve as a complementary system under specific environmental or physiological conditions [42]. The Gao-Hhe system, detected in ~48% of strains, includes domains related to haloacid dehalogenase-like hydrolases (Hhes), enzymes that are thought to interfere with phage infection by degrading nucleotides or disrupting nucleic acid metabolism [42]. While the exact mechanism remains to be elucidated, the presence of this system suggests a potential role in degrading phage DNA or interfering with its replication cycle [43].

The restriction-modification (RM) system, also present in roughly half of the strains, is a classical and well-characterized bacterial defense mechanism [44]. It consists of a restriction endonuclease that cleaves foreign (unmethylated) DNA, such as that of invading phages, and a methyltransferase that protects the host genome by methylating specific recognition sites [45]. Its continued presence in *P. salmonis* underlines its ongoing relevance as a primary and effective barrier to phage entry.

Lastly, toxin–antitoxin (TA) systems, including MazEF and SanaTA, although less frequently detected, likely serve as backup or conditional defense strategies. These systems involve a stable toxin and a labile antitoxin; under stress or phage infection, degradation of the antitoxin frees the toxin, such as MazF, an mRNA-cleaving RNase, to shut down protein synthesis, induce dormancy, or cause cell death [46]. Though energetically costly, TA systems can be highly effective in halting phage replication during advanced stages of infection or under environmental stress [27]. The co-occurrence of multiple TA systems in *P. salmonis* suggests a potential for regulatory cross-talk and context-dependent deployment of these mechanisms.

The strong positive correlation between the number of prophage regions and antiphage defense systems in the chromosomes of *P. salmonis* strains (Figure 5) suggests a co-evolutionary dynamic, where increased prophage content may exert selective pressure for the acquisition or retention of defense mechanisms [47]. This pattern aligns with previous studies indicating that bacterial genomes often balance the integration of prophages with the activation of countermeasures to prevent uncontrolled lytic cycles or superinfection [47,48]. This relationship supports the idea that bacterial genomes adapt to recurring phage exposure by expanding their defensive repertoire [48]. In contrast, the weaker correlation observed at the plasmid level may reflect the more dynamic and transient nature of plasmids, which are frequently subject to horizontal gene transfer and may carry mobile genetic elements without stable selective pressures to maintain coordinated defense. The weaker correlation observed at the plasmid level, despite being statistically significant, indicates a more variable or less coordinated association between prophage and defense system content. This discrepancy may reflect the greater mobility and functional heterogeneity of plasmids, which are often shaped by horizontal gene transfer rather than selective retention based on phage pressure alone.

In summary, in this study, we conducted an in silico discovery analysis of prophage and antiphage defense systems contained in *P. salmonis* strains available in the NCBI repository at the chromosome and plasmid levels. We showed that a high percentage of the strains, 70%, had prophage regions; each strain contained one or two regions. In these prophage regions, most of the coding regions corresponded to transposases. Similar results were revealed at the plasmid level, where 213 out of the total 331 plasmids contained in 78 strains had prophage regions; most of the plasmids contained one prophage region. At the chromosome level, transposases were the most abundant group of coding regions found in the prophage regions. These results reflect that the presence of prophages in *P. salmonis* strains could play an important role in their epidemiology. In addition, the results obtained in this study showed the presence of different antiphage defense systems contained both at the chromosome and plasmid levels, providing knowledge that could have implications for the development of phage-based therapies to increase the possibilities of SRS management in the aquaculture industry. Nonetheless, a limitation of this study lies in the current constraints of functional annotation for phage genes and proteins, as many remain poorly characterized; future improvements in annotation pipelines and reference databases will enhance the resolution and accuracy of such analyses.

## 4. Materials and Methods

A total of 79 *P*. *salmonis* genomes available in the NCBI repository were included in this study. The genomes comprise strains isolated from different species of salmonids, including *Salmo salar* (49), *Oncorhynchus kisutch* (16), *O. mykkis* (13), and *O. tshawytscha* (1). The accession codes, host, country, and year of isolation for each genomic sequence used were included in Supplementary Table S8.

### Bioinformatic Analysis

Prophage regions in the chromosomes and plasmids of *P. salmonis* strains were estimated using the PHASTEST web server [49] version 3.0. The prophage regions were classified as intact, questionable, and incomplete based on a software algorithm with default settings. Briefly, predicted prophages were identified and assigned a score based on three methods: (i) 100% of the total number of CDS in the region are linked to a known phage in the database; (ii) more than 50% of the CDS in the region are associated with a known phage; and (iii) less than 50% of the CDS in the region are related to a known phage. In the case of reaching the first method (i), a score of 150 is assigned, while for (ii) and (iii) results, the region’s score is calculated as the sum of the scores corresponding to the size of the region and the number of genes. These approaches also incorporate the identification of specific keywords related to phage components (e.g., capsid, head, integrase, plate, tail, fiber, coat, transposase, portal, terminase, protease, or lysin). Finally, the scores of methods (ii) and (iii) are compared, and the highest is chosen as the score for the region. Depending on the score, the region is assigned to one of three categories: intact (score above 90), questionable (score between 70 and 90), and incomplete (score below 70).

The presence and type of antiphage defense systems in chromosomes and plasmids of each strain were evaluated in the DefenseFinder tool version 2.0.0 [36]. This tool detects macromolecular systems using MacSyFinder, which operates using one model per system. Each model functions in two steps: (i) detection of all proteins involved in a macromolecular system by searching for homologies using HMM profiles, and (ii) a set of decision rules is applied to stay with those HMM hits that satisfy the genetic architecture of the system of interest.

The analysis of the results was carried out in the R environment version 4.4.0 [50], where the ggplot2 [51] and ggsankey [52] libraries were used to visualize results, and correlations were identified by Spearman’s rank correlation coefficient (significant thresholds were *p* < 0.05) using the *rcorr* function of the Hmisc library [53].

## Data Availability

All data related to the research results are provided in the Supplementary Materials.

## Supplementary Materials

The following supporting information can be downloaded at: https://www.mdpi.com/article/10.3390/antibiotics14060558/s1, Figure S1: Description of coding regions clustered as phage-like proteins at the chromosome (A) and plasmid (B) levels. Tables S1–S8 in excel; Table S1: Summary of the identification of prophages in *P. salmonis* genomes at the chromosomal level by PHASTEST; Table S2: Coding sequences identified in prophage regions at the chromosome level in *P. salmonis*; Table S3: Number of plasmids per strain included in this study; Table S4: Summary of the identification of prophages in *P*. *salmonis* genomes at the plasmidial level by PHASTEST; Table S5: Coding sequences identified in prophage regions at the plasmidial level in *P. salmonis*; Table S6: Chromosome-localized defense systems of *P. salmonis* strains; Table S7: Plasmid-localized defense systems of *P. salmonis* strains; Table S8: General information on the *P*. *salmonis* genomes used in this study.

## Abbreviations

The following abbreviations are used in this manuscript:

CDS	Coding sequences
SRS	Salmonid Rickettsial Septicemia
