# Supplementary material for: Know Your Enemy: Piscirickettsia salmonis and Phage Interactions Using an In Silico Perspective
Source: Antibiotics (Basel). 2025 May 30;14(6):558. doi: 10.3390/antibiotics14060558 (PMC12189261; doi:10.3390/antibiotics14060558)
Supplement: Supplementary file 1 [file antibiotics-14-00558-s001.zip › antibiotics-3597097-supplementary.pdf]

Supplementary material

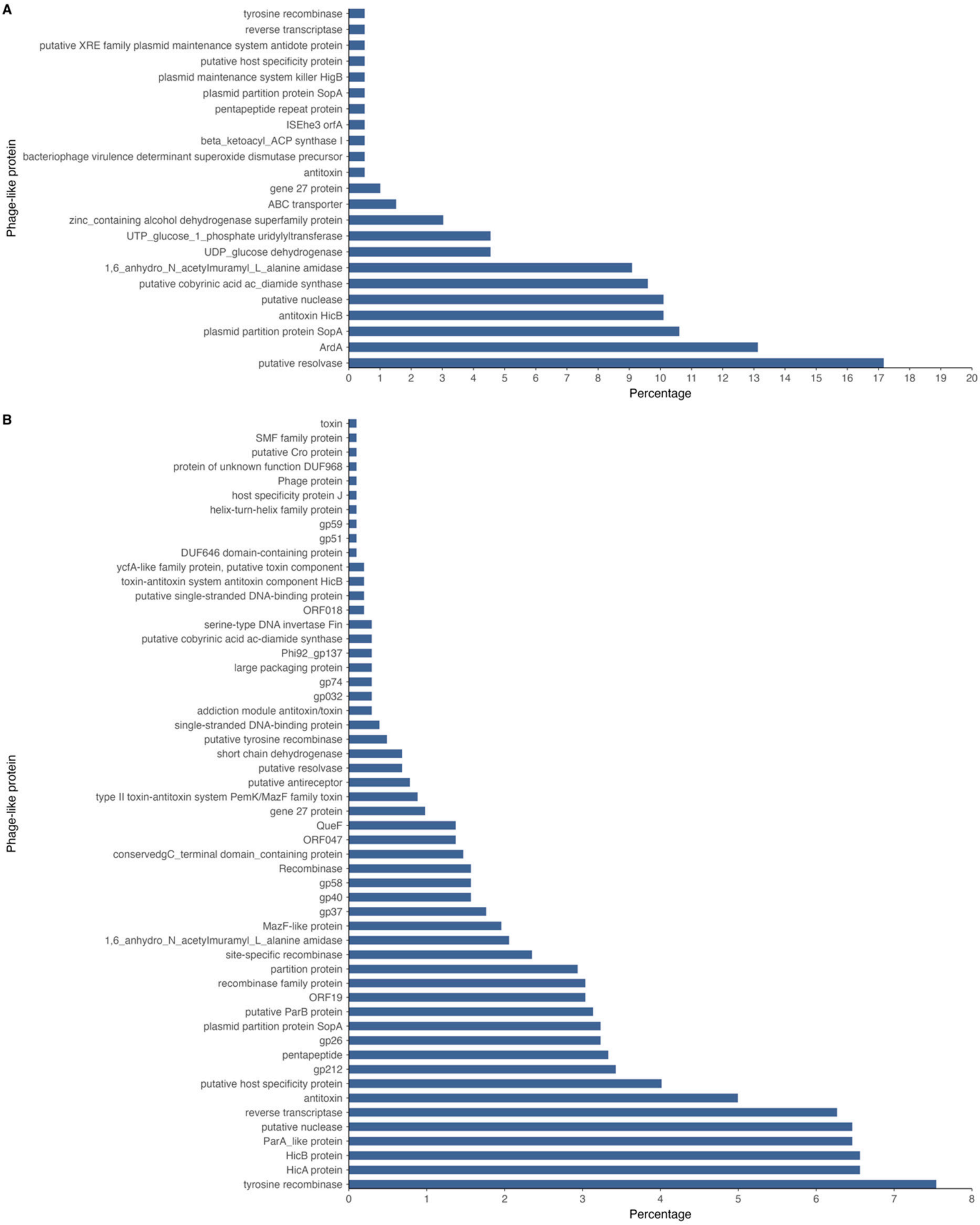

Figure S1. Description of coding regions clustered as phage-like proteins at the chromosome (A) and plasmid level (B).
